# The Effect of Name and Narrative Voice in Online Adoption Profiles on the Length of Stay of Sheltered Cats in the UK

**DOI:** 10.3390/ani11010062

**Published:** 2020-12-31

**Authors:** Chloe Rix, Mark Westman, Louise Allum, Evelyn Hall, Jessica Pockett, Camilla Pegram, Ruth Serlin

**Affiliations:** 1 Royal Veterinary College, Hatfield, Hertfordshire, London AL9 7TA, UK; chloe.rix@btinternet.com (C.R.); lallum@rvc.ac.uk (L.A.); cpegram@rvc.ac.uk (C.P.); 2 Sydney School of Veterinary Science, The University of Sydney, Camperdown, NSW 2006, Australia; mark.westman@sydney.edu.au (M.W.); jessica_marreepockett@hotmail.com (J.P.); 3 Sydney School of Veterinary Science, The University of Sydney, Camden, NSW 2570, Australia; evelyn.hall@sydney.edu.au

**Keywords:** length of stay, adoption, cats, welfare, veterinary science

## Abstract

**Simple Summary:**

A fundamental aim of shelters, pounds and other rescue facilities is to minimise the length of stay (LOS) of animals prior to rehoming, since a prolonged LOS in a shelter environment can be detrimental to behaviour, health and welfare and have a financial impact on the shelter. Previous research reveals that LOS is impacted by different factors relating to adopter preferences, to the animal (e.g., age, sex, breed, and colour), and to the shelter environment (e.g., cage placement, cage design and the provision of enrichment). This paper aimed to assess the impact of two immutable “static” factors (age and sex), and two easily changeable “dynamic” factors (cats’ names and whether the adoption description was written in the first or third person), on the LOS of cats rehomed from three charity shelters in the UK. The results demonstrated that age and sex both impacted LOS, with young cats and male cats rehomed fastest. The category of name did not affect LOS, but cats with a description written in the third person were rehomed quicker. This finding is important to shelters as it identifies a simple, no cost intervention that might save money and improve cat welfare by reducing LOS.

**Abstract:**

A prolonged length of stay (LOS) in a rehoming shelter can be detrimental to cat behaviour, health and welfare. Research shows LOS is impacted by animal signalment, behaviour and personality, whether or not previously owned or a stray, and considerations such as cage placement, cage design and the provision of enrichment. A retrospective study was undertaken at a charity organisation that rehomes surrendered and stray cats from three UK shelters. Records from 2011 to 2015, relating to 4460 rehomed cats aged between 1.0 year and 20.1 years old, were analysed to investigate factors that might affect LOS. Univariate and multivariate analysis determined the effects of name, adoption description (first person vs. third person), age and sex on LOS. The final multivariate model demonstrated that age, sex and adoption description, but not name, had a significant effect on LOS. Younger cats, male cats and cats with adoption profiles written in the third person had a significantly shorter mean LOS. Survival curves conducted using a log-rank test and time-to-event analysis, using the dates of relinquishment and rehoming, revealed that cats with a third person description had a shorter LOS. Shelters should consider writing adoption descriptions in the third person to minimise LOS.

## 1. Introduction

Animal shelters, pounds, municipal facilities and rescue organisations provide transient accommodation for stray, injured, lost and seized domestic cats (*Felis silvestris catus*) worldwide [1]. Such accommodation often involves a high number of animals being cared for by a limited number of staff with minimal resources. High density housing has been shown to be linked to increased exposure to potential pathogens including feline upper respiratory tract disease (FURTD) and dermatophytosis, as well as increased levels of stress in sheltered cats. Both of these are known to negatively impact the physical and mental health of sheltered cats, which is a major welfare concern and impediment across most rehoming facilities [2,3,4]. One study in a shelter located in north-eastern USA found that the probability of adult cats developing FURTD by day 7 after admittance was 26%, while by day 14 this probability had increased to 80% [2]. A study of a rescue shelter in Belgium found a prevalence rate of 33% for feline calicivirus (FCV) and 20% for feline herpesvirus-1 (FHV-1) over a 17-month period [3]. In the UK, the overall prevalence of FURTD (based on clinical signs) in five shelters studied over a 12 month period was 4% (60/1429) [4]. Ultimately, animals with a history of previous or current disease are less adoptable and take longer to rehome [2,5,6,7], further extending their length of stay (LOS) in the shelter, which consequently results in considerable financial implications for the rehoming facility [2,5,6,7,8].

Many factors other than health status can affect the LOS (defined as the amount of time from admission to adoption) of cats in shelters, including adopter-related factors (e.g., lifestyle, living arrangements, income and other pets), animal-related factors (e.g., age, breed, sex, coat colour, behaviour/personality, stray/previously owned) and environmental factors (e.g., shelter location, size, layout and accessibility) [9,10,11,12,13,14,15,16]. A helpful approach for shelters when considering strategies to reduce LOS is to consider whether variables within each of these three broad categories (i.e., adopter-related, animal-related, and environmental factors) are static (i.e., unchangeable) or dynamic (i.e., changeable). Categorising variables in this manner and highlighting those that are dynamic allows resources to be directed by shelters most effectively towards changes that are likely to have a real impact on reducing LOS and therefore improving animal welfare.

One of the most important adopter-related dynamic variables is the adoption fee; one survey at an Australian shelter reported that adopters of adult cats were more price sensitive than adopters of kittens, with nearly half of the adult cat adopters surveyed responding that hearing about a “low-cost“ adoption campaign was a key motivator for them adopting from the shelter [14]. Importantly, following a “low-cost” adoption, finances seemed to have minimal effect on the care and outcomes for the adopted cat, with most owners remaining highly attached to their pet 6–12 months after adoption [14]. This finding supports the notion that there is no association between the financial outlay and resources of an owner and their level of attachment to a cat [14,17,18]. It also suggests that, although potential adopters consider a number of variables in their decision-making process, such as the health status of an animal, there is a deliberate transactional decision being made that may surpass all other factors in the adoption process. One animal-related dynamic variable that has been evaluated is the presentation of animals in online adoption profiles, with a study in the USA finding that dogs standing up in photographs were rehomed faster than dogs photographed sitting down, while the presence or absence of a bandana had no impact on LOS [19]. Considering dynamic environmental factors, upper tier level cages and the presence of a toy were associated with a shorter LOS at a shelter in New York City, USA, despite the toy not having any effect on the cat’s behaviour [20]. Similarly, a study in western New York, USA, surveyed recent cat adopters to investigate the role the internet site Petfinder^TM^ played in cat adoptions by determining which variables from an animal’s online profile positively affected adoptability. This study determined that the strategic use of a toy in the adoption photo significantly correlated with a reduced LOS [21]. More recently, research looking at the effect on LOS of different patterns of language used in online Petfinder^TM^ adoption profiles concluded that adopters were more likely to respond positively to adoption profiles that used more analytical language and fewer words compared with those using more social language [22]. The author used the elaboration likelihood model (ELM) that considers how people are persuaded by different styles of advertising messages. Motivated people, who are in a position to think carefully about a decision, are more likely to be persuaded by factual, less emotional adverts, while people who are less motivated and less in a position to process a message are more likely to use simple recognition or heuristics to make a judgment [22]. This could be considered as an analysis of the “What” or content of the message and the “How” the message is conveyed.

To our knowledge, no studies have appeared in the peer-reviewed literature investigating the possible effect of the cat’s name (animal-related factor), or the narrative voice, i.e., the “Who” is telling the story in the adoption profile (environmental factor), as determined by a first person or third person approach, on the LOS of cats in shelters. The aim of the current study was to investigate whether either of these two dynamic variables affected the LOS of cats rehomed from a rehoming organisation in the UK.

## 2. Materials and Methods

### 2.1. Study Population

**Figure 1:**
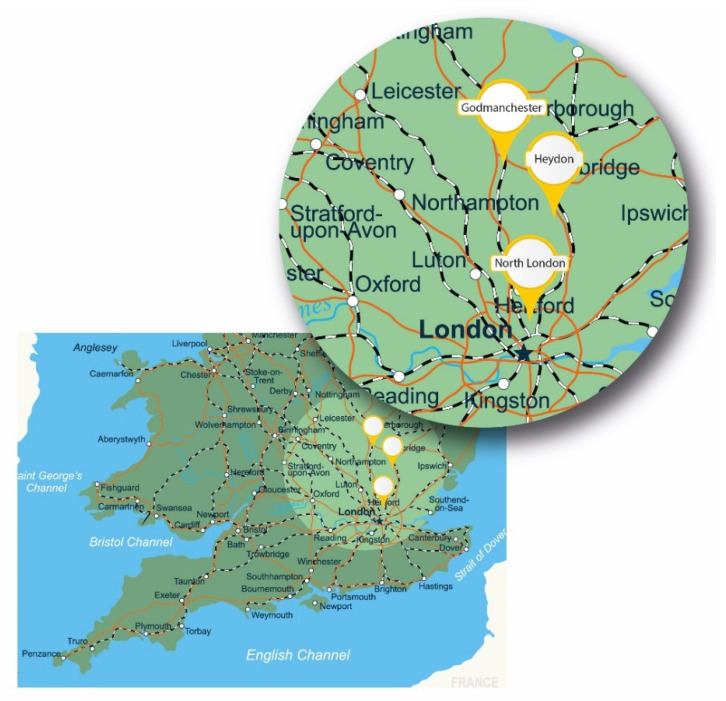
The three locations of Wood Green, The Animals Charity rehoming centres in the UK at the time of sampling. From North to South–Godmanchester, Heydon and North London (now closed)**.**

Wood Green, The Animals Charity comprises three shelters located in Godmanchester, Heydon and North London, UK (Figure 1), and rehomes surrendered or stray cats and dogs. Records pertaining to cats rehomed between November 2011 and May 2016 were retrieved in May 2016, using Anilog Animal Welfare Management Software (Innov8ive, Cheltenham, Gloucestershire, UK). Details retrieved included the cat’s age, sex, name, date of arrival at the shelter, date of departure from the shelter, LOS (days), and the adoption profile used to advertise the cat online on the Wood Green, The Animals Charity website. The majority of cats also had this profile on their cage.

The age of the cat was either provided by the previous owner (surrendered cats) or estimated by shelter staff using dental wear and physical traits such as size and coat greying (stray cats). Kittens and cats less than one year of age were excluded from the study, since several previous studies have demonstrated cats of this age to be the fastest to rehome, thereby having the potential to skew results [6,8,9,11,15,23,24]. Other animals were excluded due to data errors such as missing data fields. For the purposes of the study, cats were grouped into one of three categories, based on their estimated age at adoption: young adults (1 to <4 years), adults (4 to <7 years) and seniors (≥7 years). All cats included in the study were neutered. The breeds of cats were not captured during the data retrieval process.

Cats were managed similarly across the three sites of the same charity. The Godmanchester centre has facilities to house up to 70 cats, Heydon—40 cats and North London—up to nine cats. At the time of the study at each site, the cats underwent a veterinary examination within three days of arrival and were vaccinated and treated with anti-parasitic medication during this check. The housing pens present at the three different sites were similar in terms of design and size: Heydon and North London used pens made of unplasticized polyvinyl chloride (UPVC) while the Godmanchester site used wooden pens that were slightly smaller. In all three centres, individual cats were housed in single pens with an enclosed outside run. Paired cats were housed in two adjacent pens and the barrier between the two runs removed. This effectively doubled the space, whilst keeping the integrity of the internal wall between the pens. Rarely, larger groups (i.e., more than two cats) were accommodated by similar adaptations. All pens contained a water bowl, a bed, litter trays, and toys and scratching posts for enrichment. The cats were routinely fed twice daily with proprietary cat food either from bowls, enrichment feeders or were scatter fed. Each rehoming site provided the same enrichment and resources for the cats. Volunteers at each site would attend and interact with the cats two to three days a week but had no contact with potential adopters.

At the time of sampling, social media advertising was not prioritised and fewer than 20 cats per year were advertised on websites other than the main Wood Green, The Animals Charity web page. Approximately 40 cats were advertised at off-site charity events away from the shelters. A small fostering program also existed that mainly involved a handful of queens and kittens which were not involved in the current study.

Human ethics approval was given by the Royal Veterinary College (Protocol Number 2016/U135).

### 2.2. Name of the Cat

**Figure 2:**
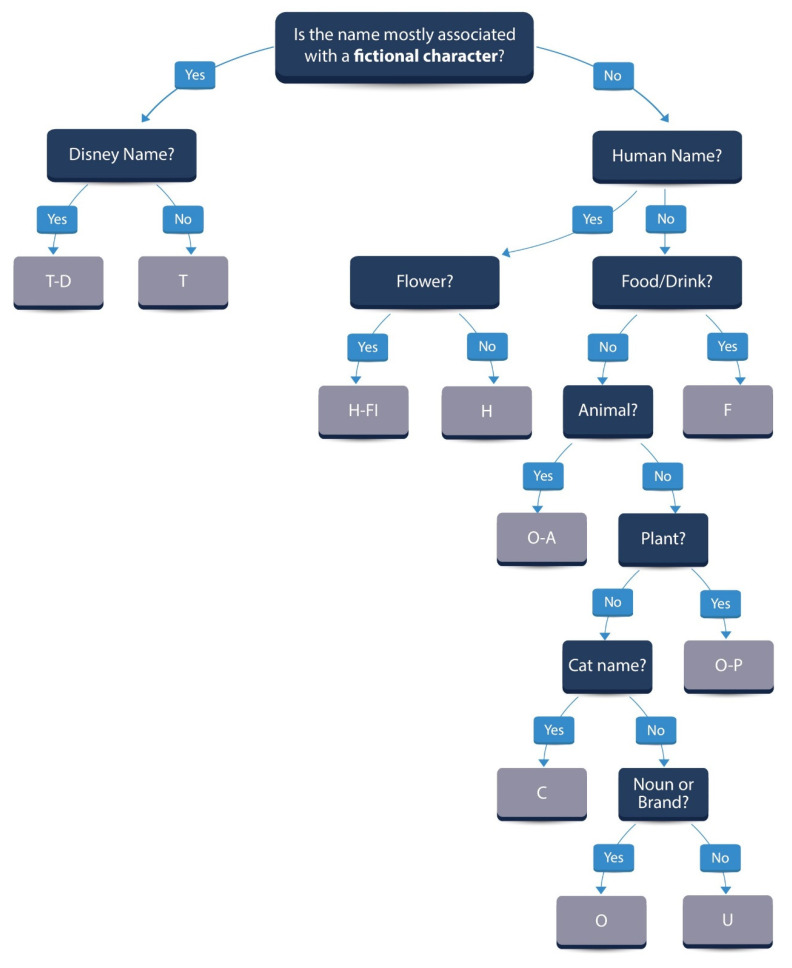
The algorithm used for retrospectively categorising cat names. Ten categories were created: animal names (O-A), cat names (C), food/drink (F), human names (H), flowers (H-Fl), plants (O-P), fictional character names (T), Disney^TM^ names (T-D), other/miscellaneous names (O) and unusual/uncommon names (U).

A naming algorithm was inductively created through repeated analysis of the data by the primary author (C.R.), with ten categories (eight major, two minor) created to allow statistical analysis (Figure 2). Cats were then retrospectively allocated to a category by the primary author (C.R.). The eight major name categories were animal names (O-A), cat names (C), food/drink (F), human names (H), flowers (H-Fl), plants (O-P), fictional character names (T) and Disney^TM^ names (T-D). Names that did not fit into one of these eight main categories were placed into one of two minor categories: other/miscellaneous names (O) if they were nouns or brands, or unusual/uncommon names (U) if they were not. The same author (C.R.) also recruited ten independent observers to assist with categorising names that did not easily fit into one of the ten categories. These observers can be considered a convenience sample and consisted of student peers, friends, family and members of staff at the Royal Veterinary College. Answers from these 10 respondents were blinded, and the most common answer given was selected. In total, 99 cats had names that were difficult to categorise and required additional observer input.

Indicative examples of cat names and the categories to which they were allocated are given in Table 1.

### 2.3. Description of the Cat (Narrative Voice)

The adoption profiles were retrospectively assigned a value of 1 or 3, by one of the primary authors (C.R.) according to whether the narrative voice was the first person (e.g., “Hi my name is Bear, I am a sweet boy and I would love my own home…”) or in the third person (e.g., “Tiger is a handsome chap who came to us as a stray…”).

### 2.4. Statistical Analysis

Initial statistical analysis was conducted in GenStat^®^ (v.18, VSN International, Hemel Hempstead, United Kingdom). The outcome variable data (i.e., LOS) were assessed for normality using a Shapiro–Wilk normality test, which showed that the data were not normally distributed and required log_e_ transformation for analysis in order to compare mean LOS between explanatory variables. Univariate analysis using a linear mixed modelling approach was then performed by testing each explanatory variable (sex, age, name and adoption description) on its own against the outcome. All explanatory variables with *p* < 0.25 in the univariate model were retained for multivariate analysis. Explanatory variables with *p* < 0.05 in the final model were considered significant, and residual plots were performed to check that the final multivariate model was a good fit and that this model was appropriate. The F statistic, numerator of degrees of freedom (n.d.f), denominator degrees of freedom (d.d.f.) and *p* value are reported for each analysis, with the standard error reported for significant variables.

A Kaplan–Meier survival (i.e., time-to-event) curve was constructed to describe the time to rehoming from shelter admission in cats with different adoption descriptions (i.e., first person or third person). Log-rank tests were used for comparisons between categories [25].

## 3. Results

### 3.1. Study Population

Of the 8628 records retrieved, 4460 rehomed cats were analysed for the study. In total, 2583 cats were excluded due to age (<1 year), 1291 cats were excluded due to having no recorded adoption profile, and 294 cats were excluded for not having a recorded date of birth.

The median age of the 4460 cats analysed was 4.0 years old (range 1.0–20.1 years; interquartile range 2.0–7.1 years). In total, 47% (2075/4460) of cats were classified as young adults (1–<4 years), 26% (1146/4460) were adults (4–<7 years), and 28% (1239/4460) were seniors (≥7 years). The overall male to female ratio was 0.75:1 (1903 males to 2557 females) (Table 2). In total, 1789/4460 adoption profiles (40%) had been written in the first person and 2671/4460 had been written in the third person (60%). More than three-quarters of adopted cats (3382/4460; 76%) were rehomed within 39 days (mean LOS 32.2 days, median LOS 18.0 days, range 0–351 days, interquartile range 10–39 days).

### 3.2. Univariate Analysis

Each explanatory variate was tested on its own against the outcome (LOS). Age, sex and adoption description were significant with univariate analysis (*p* < 0.001) (Table 3) and were therefore carried into a multivariate analysis. The category of name (Table 4) was not found to be significant with univariate analysis (*p* = 0.198) and was therefore dropped from the multivariate analysis.

### 3.3. Multivariate Analysis

Each of the three explanatory variables tested (age, sex and description) retained their significance in the multivariate model (*p* < 0.001) (Table 5). Interactions were tested between the variables to assess if any were significant, with none found (Table 5). Thus, the final model used was Length of Stay~Age + Sex + Description. Residual plots indicated that the multivariate model was a good fit, suggesting that this model was appropriate.

**Figure 3:**
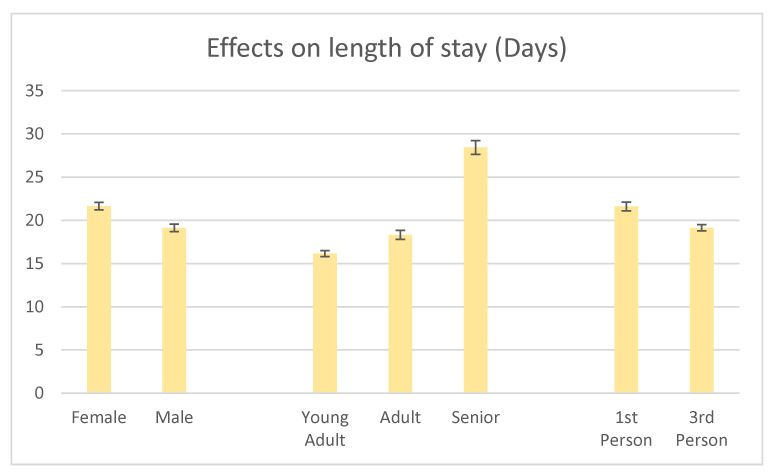
Results from the multivariate analysis. Sex, age and narrative voice of the adoption profile (first person vs. third person) were all found to significantly affect mean LOS (*p <* 0.001). Standard error bars are shown.

In conclusion, age, sex and narrative voice description had a significant effect on mean LOS (*p* < 0.001) (Figure 3). There was a significant difference in LOS between all age categories. Young adult cats (1–<4 years) had the shortest mean LOS (16.15 ± 0.36 days), followed by adult cats (4–<7 years) (18.32 ± 0.53 days), with senior cats (≥7 years) having the longest mean LOS compared with both other age groups (28.45 ± 0.80 days). Female cats had a greater mean LOS than male cats, with a difference of more than two days (21.65 ± 0.43 vs. 19.13 ± 0.44 days). Cats with descriptions written in the first person had a longer mean LOS of more than two days compared to those written in the third person (21.61 ± 0.50 vs. 19.14 ± 0.36 days).

**Figure 4:**
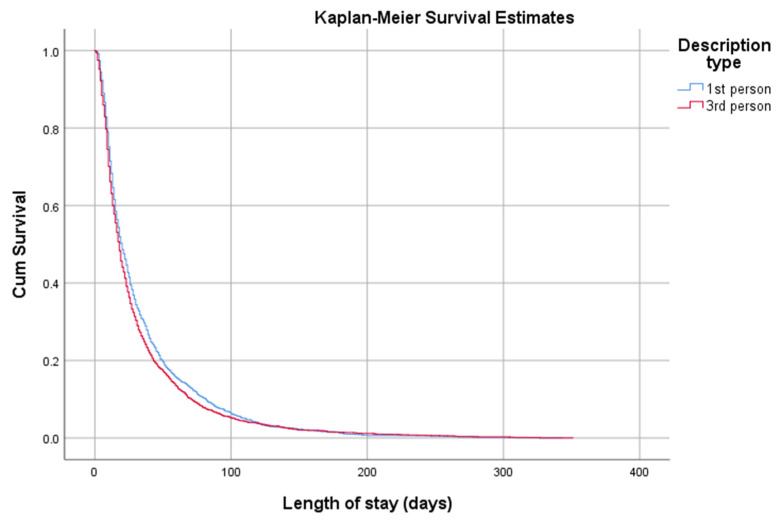
Kaplan-Meier survival curve of time-to-rehoming for cats according to the narrative voice of the adoption profile (first person vs. third person). Survival time represents the time from shelter admission to rehoming (LOS).

Comparing the time-to-event from shelter admission to rehoming, there was a significant difference in the survival curves between the first person and third person description types (log-rank test, *p* = 0.003) (Figure 4).

## 4. Discussion

The current study focused on two dynamic factors (one animal related, one environmental) that might impact the LOS of shelter cats, namely the cat’s name and a specific element of its adoption description. These two factors differ from static (i.e., immutable) adopter-related, animal-related, and environmental factors that shelter staff are unable to change. Previously owned cats will already have a name at relinquishment, although this can be changed if desired, and stray cats are named by shelter staff. The narrative voice is chosen by the shelter staff when they advertise the cats online or place the adoption details on the cage in the shelter. This study found that the narrative voice used in the adoption profile of cats rehomed from Wood Green, The Animals Charity, which is comprised of three shelters within the UK, significantly influenced a cat’s LOS, with cats advertised with an adoption profile written in the third person having the shortest LOS. This is, to our knowledge, the first time that the effect of narrative voice has been investigated.

Considering the effect of persuasive language in human medicine, research shows that, in contrast to our findings, a narrative or story telling approach in messaging is more likely to affect positive change in patient behaviour than one based on an analytical approach. One study reported that a first person narrative was more than twice as likely to have a positive effect than a third person narrative [26]. This finding is echoed in the educational literature, with teachers told a narrative in the first person found to be more likely to change their opinion about a pedagogical intervention than teachers who read an analytic, statistic-driven informative article written in the third person [27]. The inference is that first-hand narrative is more powerful than second-hand, and both are more powerful than “abstract information” [27].

In contrast, a recent analysis of over 180,000 online profiles of pets in shelters awaiting adoption, looking at the content and structure, i.e., the “What?” and the “How?” of the adoption profiles, revealed that potential adopters of pets preferred a more analytically written profile. Those using more “markers of analytic thinking” in their content, compared with a more emotive, social, narrative content, were associated with a reduced LOS. More humanising references were also found to be dispreferred by adopters. Instead, a high rate of ingestion words (e.g., chew), detailing objective descriptions about an animal’s actions and experiences, corresponded to a shorter LOS [22].

Looking at advertising through the lens of literary theory, Stern [28] discussed the effects of presenting a story from a first person vs. a third person perspective. She posited that a first-person narrative creates intimacy and humanises the story, in contrast to a third person narrative, which is more likely to be used to convey information and is thus perceived to be more objective. Given the fact that potential adopters are more likely to choose a cheaper adult cat [14] and they prefer an analytical approach in the adoption description [22], the finding that the third person narrative results in a shorter LOS makes sense in as much as potential adopters feel this is a more analytical approach to advertising the cat. Results from market research conducted in Poland support this conclusion, with rational, non-emotive advertising leading to more positive attitudes towards products and higher purchase intentions than emotional advertising [29]. A recent study on personality adjectives used in the descriptive text of dogs in Australia found that the presence of some breed-related adjectives and the absence of others also influenced LOS [30].

Our study on the adoption information of cats rehomed from the Wood Green, The Animals Charity shelters does not consider the “What?” or the “How?” of the adoption information, but the “Who?”, finding that using the third person approach was associated with a decreased LOS. It seems that potential cat adopters behave more as consumers of advertising messages than recipients of healthcare messages [26] and stories about educational practice [27].

Consideration of the “What?” and the “How?” of adoption information from the Wood Green, The Animals Charity shelters could be the subject of further analysis using the ELM framework as proposed by Markowitz [22]. The adoption profile also might be considered to be a “genre” as defined in the Systemic Functional Linguistic (SFL) approach to language analysis, which considers how language is used to “get things done” in a given context [31]. Using this latter approach, the adoption profiles would be evaluated for elements of linguistic genre. Analysis would consider how readers make sense of the text through the interaction of the text and its context, considering the function of elements in the text; e.g., whether it is “informing”, “inviting” or “instructing” the reader, together with the analysis of the “register” or the formality of the language and how the relationship between the writer and reader is managed. Results from a SFL analysis could explore the possible effect of the different elements of the adoption description on LOS. The results of this analysis could be used to give more empirical advice on how best to structure an adoption profile.

Future research, which was also not possible in the current study due to the retrospective design, should attempt to investigate how it was decided that an adoption description would be written in first or third person. It is possible that it was solely due to the personal preferences of an individual staff member, and this may have included unconscious personal bias towards particular cats. For example, cats perceived as being “more difficult” than others may have been given an adoption description written in the first person narrative, thereby skewing the results in favour of third person narratives. Furthermore, it would be also useful in the future to investigate if the findings from the current study are repeatable at rehoming organisations situated in different areas of the UK and internationally to determine if geographical and cultural differences influence a cat’s adoptability due to differing perspectives on the importance of particular traits. The data retrieved did not contain the details of the cats’ locations at different shelters and so local shelter management details could not be explored further.

Whilst all the adoption profiles appeared online, it is noted that this description will also have been used on the “cage card” in the cattery in the vast majority of cases. Arguably, the potential adopters will have already formed an impression of the cat before they arrived onsite, but further studies could control for this variation in the presentation. A small group of supporters would take a few of the descriptions to charity events to advertise the cats there. The total number of cats for which this occurred is around 40, and therefore not likely to have skewed the results for the over 4500 cats analysed.

Results from the current study demonstrated that the category of name did not affect the LOS of cats, implying that UK adopters either do not take name into consideration when choosing a cat, knowing it can be changed following adoption, or that name is less important than other factors. A survey of adopters conducted at a shelter in Australia found that the three most common considerations when adopting a cat were the suitability of the adopter’s accommodation, the cat’s personality, and the adopter’s lifestyle [14]. Given that a cat’s personality is an important consideration for adopters, this finding from the current study suggests that cat adopters do not associate a cat’s name with its personality, in contrast with the way dog adopters feel that the dog’s name reflects its personality [14,32].

The analysis of our two static variables, age and sex, revealed that they both had a significant effect on LOS. Young adult cats (1–<4 years) had the shortest LOS, followed by adult cats (4–<7 years) and senior cats (≥7 years). This finding is similar to the majority of previous studies, which consistently report a trend of decreased adoptability with increasing age of the cat [8,20,23,24,33,34]. In addition to this trend, one study found, through the surveying of adopters, that one of the most important considerations for potential adopters when choosing a companion animal from a shelter was age [15]. However, in a study conducted across three shelters in the Czech Republic, contradictory findings were reported with adult cats (5–10 years) rehomed faster than kittens, juveniles and young adult cats [9]. With regards to sex, male cats had a shorter LOS compared to female cats, which also followed the general trend reported in other studies [20,24,33]. Brown and Morgan et al. (2015) attributed this finding to behavioural differences between male and female cats, with potential adopters perceiving male cats to be more approachable and playful [24]. There are other studies, however, that have reported no difference in the LOS between male and female cats [8,9]. Both of these contrasting reports had a predominance of female cats within their study populations, which may account for the different results. In view of these inconsistent findings, it may also be possible that there are additional factors influencing the preferences of potential adopters such as cultural or geographical factors.

A limitation of the current study was that many names fit into multiple categories, despite the naming algorithm used (Figure 2), and therefore there was some subjectivity with the final categorisation. For example, “Robin” could have been categorised as an animal name after the bird, a human name, or a fictional character name after “Robin Hood” or Robin in “Batman”; “Tyrion” and “Khaleesi” could have been considered unusual/uncommon names, or fictional character names from the television series “Game of Thrones”. To overcome this challenge in future studies, adopters could be surveyed at the time of adoption to identify their perception of the cat’s name, whether it influenced their decision to adopt a particular cat, and whether they planned to retain the cat’s name following adoption.

A second limitation of this study was in relation to the scarcity of information obtained during the initial data capture at the time of admission of the cats, and in some cases, the failure to record known variables on the database. Breed is a variable that has been shown to have a significant effect on the LOS of cats in shelters. It has been consistently reported in previous studies that purebred cats have a shorter LOS in comparison to non-purebred cats [2,8,13,24]. However, as the breed information was not retrieved from the database, it was not possible to consider the effects of breed in this study. Similarly, housing has been shown to play a significant role in accelerating the rehoming of cats, with high quality housing allowing them to display natural behaviours, improving their mental wellbeing and increasing the number of positive interactions with potential adopters [35]. Within the three shelters analysed in this study, the cats were usually housed individually or in bonded pairs. Although the housing was similar at each of the sites, specific housing data for cats in the present study was incompletely recorded and was therefore unable to be considered as a potential explanatory variable during the data analysis. To our knowledge, there have been no studies which have investigated the effect of single vs. paired housing in shelters on LOS. Due to the known impacts that type of housing can have on the LOS of cats in shelters, future studies should consider and investigate the significance of this variable on LOS.

Other variables of importance that were also not considered in the current study included the behaviour of the animal, the provision of toys within the animal’s cage, the cost of adoption, geography/culture of adopters, and the photo used of the cat on the website (if present). All of these variables should be investigated in future studies to better understand their effect on the LOS of cats in rehoming facilities. The behaviour of a cat is considered to be one of the most important considerations for potential adopters, with several studies reporting that behavioural traits rank higher than those of physical appearance [10,11,12,15,20]. Previous studies have reported that active cats and cats who appeared “friendly”, “happy”, “playful” and “relaxed” were viewed for a significantly longer period than less active cats and were deemed as being more attractive to potential adopters [11,12]. The same studies branched into semiotics, considering the impact of the placement of toys in the animal’s cage, creating the perception that these cat’s had “playful” and “relaxed” personalities [11,12]. Since the current study was retrospective in nature, and no owner questionnaires were performed, future studies should consider surveys to gain insight into the adopter’s impression of their cat’s behaviour upon viewing and whether it was a deciding factor for adopting a particular cat. Determining which cats are viewed as having undesirable personality traits by potential adopters will enable shelters to intervene in order to make these cats more adoptable, for example introducing them into group-housing or providing them with a visually aesthetic toy. In addition to this, the retrospective nature of this study meant that the potential relationship between a cat’s name and their perceived personality was unable to be considered. Future studies should attempt to investigate this assumption to determine whether adopters perceive a possible relationship between the name given to a cat and its personality traits.

The adoption fee and the potential impact of changes in the fee, such as low-cost adoption campaigns, was another variable that was not recorded by the shelters analysed in this study. As previously mentioned, “low-cost” adoption campaigns have been found to be a key motivator in the adoption of an animal for some adopters [14]. Future studies should take the adoption fee into consideration to determine if costs are the superior motivator for potential adopters, over other variables including those associated with the physical and behavioural traits of the animal, the adoption description, and how they are presented and marketed on associated websites.

Season has also been found to be an influencing factor on the adoptability of cats within shelters. One study found that adoption rates increased following the breeding season (spring/summer) [9]. The effect of season on LOS was not considered in the current study as it was focused on the dynamic factors associated with the cat’s name and adoption description. Future studies with the aim to investigate the adoptability of cats in shelters should consider seasonality to determine if adoptability increases at particular times of the year, for example in winter when people tend to spend more time indoors. If a seasonal trend is discovered across shelters, rehoming organisation could introduce strategies to combat low-season adoptions such as “low-cost” adoption promotions and increased advertising.

## 5. Conclusions

The results of this study demonstrated that the dynamic and easily changeable narrative voice of the adoption profile, but not the category of name, affected the LOS of cats rehomed from three shelters in the UK. Similar shelters are therefore advised not to spend time or effort choosing names, thinking it will make cats more adoptable, and instead focus on writing adoption profiles using a third person narrative style to fit with a more informative, analytical approach. More research needs to be performed to further characterise the preferences of animal adopters from shelters, relating to both static and dynamic factors, but also to evaluate how the fields of study such as semiotics and applied linguistics can help shelters to present their cats in the best light to potential adopters.

## Figures and Tables

**Table 1:** Examples of different cat names and how they were categorised.

Name Category	Examples of Names
Animal names (O-A)	Tiger, Monkey, Cougar, Bunny, Squid
Cat names (C)	Kitty, Tabby, Felix, Tibby, Sparkle
Food and drink (F)	Treacle, Saffron, Peaches, Biscuit, Pumpkin
Human names (H)	Bridget, Alfie, Sophie, Claude
Flowers (H-Fl)	Daisy, Lily, Jasmine, Rose, Poppy
Plants (O-P)	Bark, Maple, Tumbleweed, Shamrock, Bramble
Fictional names (T)	Zippy, Mork, Mario, Dobby, Katniss
Disney^TM^ names (T-D)	Nemo, Stitch, Bambi, Elsa, Mowgli
Other/miscellaneous names (O)	Silver, Bow, Switch, Pretty, Clouds
Unusual/uncommon names (U)	Pop, Zizzle, Vonnie, Meep, Bead

**Table 2:** Summary of the signalment of 8628 cats rehomed from Wood Green, The Animals Charity between November 2011 and May 2016, including the 4460 cats analysed for the study. In addition to the 2583 kittens excluded from the study, 1291 cats were excluded due to having no recorded adoption profile and 294 cats were excluded for not having a recorded date of birth.

Age Category	Male	Female	Total	Included/Excluded
<12 months of age	1265	1318	2583	Excluded
Young adults (1–<4 years)	870	1205	2075	Included
Adults (4–<7 years)	516	630	1146	Included
Seniors (≥7 years)	517	722	1239	Included

**Table 3:** Summary of the results from univariate analysis. All explanatory variables (age, sex and adoption description) were significant and carried into a multivariate analysis (*p* < 0.001), except for the name category (*p* = 0.198). Description = narrative voice (i.e., first person vs. third person). Refer to Table 4 for a numerical summary of the data by name category.

Variable	F Statistic	DF	*p*
Age	135.49	4453	<0.001
Sex	18.25	4454	<0.001
Description	17.90	4454	<0.001
Name Category	1.37	4446	0.198

DF—degrees of freedom.

**Table 4:** Summary of results from retrospective name categorisation. Ten categories of names were created: animal names (O-A), cat names (C), food/drink (F), human names (H), flowers (H-Fl), plants (O-P), fictional character names (T), Disney^TM^ names (T-D), other/miscellaneous names (O) and unusual/uncommon names (U). Category of name was not found to significantly affect the mean length of stay (LOS) in the univariate model (*p* = 0.198).

Name Category	O-A	C	F	H	H-Fl	O-P	T	T-D	O	U	Total
Number of cats	50 (1%)	974 (22%)	317 (7%)	1872 (42%)	93 (3%)	34 (1%)	269 (7%)	170 (4%)	202 (5%)	479 (11%)	4460
Sex % (M/F)	54/46	39/61	37/63	44/56	0/100	18/82	61/39	42/58	45/55	46/54	43/57
Median age (years)	4.0	4.1	3.1	4.1	4.0	3.0	4.0	3.2	3.1	4.1	4.0
	(1–15.8)	(1–20.1)	(1–15.2)	(1–18.8)	(1–16.1)	(1–14.1)	(1–17.7)	(1–14.3)	(1–15.1)	(1–15.8)	(1–20.1)
Median LOS (days)	21	18	16	19	19	19	17	19	18	19	18
	(2–165)	(1–317)	(3–326)	(0–351)	(1–225)	(4–112)	(1–247)	(2–196)	(2–171)	(2–343)	(0–351)

**Table 5:** Summary of the results from the multivariate analysis, including the analysis of potential interactions. Description = narrative voice (i.e., first person vs. third person).

Variable	Wald Statistic	n.d.f.	F Statistic	d.d.f.	*p*
Age	42.46	2	135.41	4451	<0.001
Sex	13.77	1	17.63	4451	<0.001
Description	29.84	1	16.68	4451	<0.001
Sex*Age	5.74	2	2.87	4450	0.057
Sex*Description	2.11	1	2.11	4452	0.147
Age*Description	2.16	2	1.08	4450	0.338
Sex*Age*Description	2.88	2	1.44	4444	0.237

n.d.f.—numerator degrees of freedom; d.d.f.—denominator degrees of freedom; *—potential interaction between two variables being tested.

## Data Availability

Data is sensitive to the charity.
